# Patterns of Adverse Childhood Experiences from a Longitudinal South African Community Sample: A Latent Class Analysis

**DOI:** 10.1007/s42448-025-00231-5

**Published:** 2025-08-28

**Authors:** Christina Thurston, Aja Louise Murray, Hannabeth Franchino-Olsen, Franziska Meinck

**Affiliations:** 1 Department of Social Work, Chrystal Macmillan Building, The University of Edinburgh, 15a George Square, Edinburgh, EH8 9LD UK; 2 Department of Psychology, University of Edinburgh, Edinburgh, UK; 3 College of Public Health, The Ohio State University, Columbus, OH USA; 4 School of Public Health, University of the Witwatersrand, Johannesburg, South Africa; 5 Faculty of Humanities, North-West University, Potchefstroom, South Africa

**Keywords:** Adolescents, South Africa, Latent class analysis, Adverse childhood experiences, HIV

## Abstract

South African children have a high risk of experiencing multiple adverse childhood experiences (ACEs) compared to children living in high-income countries. In a low- and middle-income context with high levels of deprivation and crime, we should consider expanded ACEs (ACE-E) that are contextually sensitive, alongside conventional ACEs (ACEs-C). Latent class analysis was used to explore patterns of 14 ACEs measured in a sample of 3,401 South African adolescents (56.42% girls). Data were collected in 2010/2011 and 2011/2012. We assessed the association between class membership and demographic covariates of poverty, location (rural vs. urban), gender, age, and province. 5 classes emerged based on model fit indices and theory: “Low ACEs,” “High Parental AIDS-Affectedness and Parental Death,” “High Parental AIDS-Affectedness and Parental Sickness,” “Moderate Multi-Type Abuse,” and “Highest Multi-type ACEs.” ACEs-E played a stronger role in shaping latent classes than ACEs-C. Compared to the low ACE class, adolescents in other classes lived in greater poverty, were older, and more likely to be girls. Children in “High Parental AIDS-Affectedness and Parental Death” were more likely to be in the Mpumalanga province and children in “Moderate Multi-Type Abuse” were more likely to be in Western Cape. South African adolescents are at risk of multiple ACE exposure and contextually sensitive ACEs mattered most when characterising patterns of ACEs. Research should continue to focus on person-centred approaches to ACEs for understanding the complexity and multiplicative effects of ACEs in low-resource settings.

**Supplementary Information:**

The online version contains supplementary material available at 10.1007/s42448-025-00231-5.

## Introduction

The term “adverse childhood experiences” is used to describe stressful and potentially traumatic life events that happen in childhood, between 0 and 17 years old (Centers for Disease Control and Prevention (CDC), 2019). Occurrences traditionally recognised as ACEs in Felitti et al.’s (1998) seminal ACE study fall under the three umbrella categories of neglect (emotional; physical), abuse (emotional; physical; sexual), and household dysfunction (parental substance abuse; parental mental illness; parental separation or divorce; mother treated violently; criminal behaviour in household). The original ACE study sample was mainly white and middle-class and the questions that measured the 10 original ACEs (often referred to as Conventional ACEs (ACEs-C) in literature) are still utilised in large, nation-wide studies today such as the Behavioural Risk Factor Surveillance System (CDC, 2022). However, the exclusion of locally relevant forms of childhood adversity has led researchers to question the adequacy of ACEs-C in samples with above average rates of poverty and crime and whether these originally measured experiences are as contextually relevant for populations of varying ethnicities and levels of socioeconomic status in the USA, as well as populations in low- and middle-income countries (Giovanelli & Reynolds, 2021).

Since the original ACE study (Felitti et al., 1998), a significant body of work has expanded the conceptualisation and measurement from ACEs-C, with occurrences such as peer violence, discrimination, and witnessing or experiencing community violence now recognised in childhood adversity measures such as the Juvenile Victimisation Questionnaire (Finkelhor et al., 2005a, b) and the Adverse Childhood Experiences- International Questionnaire (World Health Organization, 2018). These other experiences have been referred to as Expanded ACEs (ACEs-E) in literature (Cronholm et al., 2015; Karatekin & Hill, 2019).

Both ACEs-C and ACEs-E are repeatedly recognised as major risks to global public health concerns (Bellis et al., 2019; CDC, 2023; Ceccarelli et al., 2022; Hughes et al., 2017; Monnat & Chander, 2015). These adversities have been consistently linked to poor mental health (Sahle et al., 2022), physical health (Hales et al., 2023) and health-harming behaviours across the life course (Bellis et al., 2023). They also have demonstrated a negative impact intergenerationally (Su et al., 2022). Evidence in low- and middle-income country (LMIC) settings and/or low-resource contexts confirm significant associations between ACEs and poor health outcomes (Blum et al., 2019; Coêlho et al., 2016; Giovanelli & Reynolds, 2021; Kidman et al., 2020; Ramiro et al., 2010). A wealth of studies have also confirmed the importance of incorporating ACEs-E when exploring prevalence, patterns of ACEs, and their associations with deleterious outcomes in these contexts (Ferrajāo et al., 2022; Franchino-Olsen et al., 2024; Ismayilova et al., 2016; Muñoz et al., 2024; Naidoo et al., 2024).

### Cumulative Risk Versus Latent Class Analysis

Most extant research on ACEs has concentrated on cumulative ACE scores that sum the number of ACEs one has experienced. This approach views the potential negative effects of ACEs through an additive lens. Increasing literature has focused on comparing outcomes for different ACE scores, mainly differentiating people who have experienced 0–3 ACEs versus 4 + ACEs (e.g., Bellis et al., 2014; Felitti et al., 1998; Hughes et al., 2017; Witt et al., 2019). Literature investigating outcomes based on cumulative ACE scores concurs that those who have 4 or more ACEs have a significantly higher risk for a multitude of poor outcomes compared to people who have experienced 0–3 ACEs (Meehan et al., 2022).

Whilst cumulative ACE scores associating 4 + ACEs with worse outcomes have been used in large-scale, nationally representative survey research such as the English national household survey of ACEs by Bellis and colleagues (2014), analyses that use additive ACE scores infer each adversity has equal importance in the development of the measured outcome and neglects that some childhood adversities may lead to worse outcomes than others (Lacey & Minnis, 2020). Furthermore, research exploring linear, additive effects of ACEs, and poor outcomes may neglect the possibility that the ACEs may co-occur and that the effects of single adversities may combine and interact to impact outcomes (Brown et al., 2019).

Person-centred approaches, such as latent class analysis (LCA) aim to identify unmeasurable or naturally underlying subgroups within a population based on individual’s responses across multiple variables (Lanza & Rhoades, 2013; Vermunt & Magidson, 2004). When applying this statistical procedure to ACEs research, LCA can group participants into latent profiles of adversities or subpopulations based on the participants’ measured ACE exposure (Lanier et al., 2018). Most notably, LCA acknowledges the possibility of co-occurring ACEs and does not assume the equal importance of ACEs on measured outcomes (McLafferty et al., 2015). Consequently, LCA provides a solution to the limitations of analyses using cumulative “ACE scores” and those that assume single ACE effects (Shin et al., 2018). Whilst research confirms ACEs co-occur (Brown et al., 2019; Lanier et al., 2018), more research is needed to understand the specific combinations of ACEs that co-occur in different contexts. Enhancing our understanding of ACE patterns can support us to better inform social policy and guide both prevention and intervention research.

### The South African ACE Setting

Research has posited people residing in low-and middle-income countries (LMICs) may have a heightened risk of ACEs compared to those living in high-income countries; an assertion often attributed to higher rates of poverty and co-occurring socio-environmental factors (Ramiro et al., 2010). In South Africa, rates of extreme poverty have been exacerbated by Covid-19 (Wills et al., 2023). In addition, South Africa has a higher prevalence of violence against children (Meinck et al., 2016), a larger orphan population (Hall, 2024) and more prevalent incidents of fatal child abuse compared to some high-income countries like the United Kingdom (Abrahams et al., 2024; Mathews et al., 2013). These disparities underscore the importance of why ACE research conducted in high-income countries cannot be universalised to LMIC countries such as South Africa.

Furthermore, children in families with lower socioeconomic positions face disproportionately more adversity than their peers who live in families with higher socioeconomic positions (Institute of Health Equity, 2015) and experiencing poverty and ACEs results in children being at a higher risk for lifelong poor physical and mental health outcomes (Macedo et al., 2018; Plagerson et al., 2011).

Within the South African context, the HIV/AIDS epidemic is also a pertinent consideration when looking at contextually sensitive ACEs, with an estimated 8.45 million people (around 14% of the population) living with HIV in 2022 (South African Government, 2024). HIV/AIDS is an illness that has been particularly stigmatised (Mahajan et al., 2008), and such stigma may be one of the reasons that South African children living in households impacted by HIV/AIDS—including AIDS-orphanhood—have been found to experience negative outcomes similar to those associated with more conventional ACEs, including showing a greater risk of psychological distress (Cluver & Gardner, 2007), and higher levels of PTSD, anxiety and depression compared to non-orphans or other-orphans (Cluver et al., 2012).

Overall, South African children consequently may be more at-risk of developing long-term consequences of widespread childhood adversities that are not as prevalent in high-income countries (Breen et al., 2015) and thus the way in which these ACEs cluster together, whether ACE-C or ACEs-E matter more in a South African context, ACE prevalence rates, and the sociodemographic variables that may influence risk of exposure to ACEs are deserving of further research and will be investigated in this current study.

 To the best of our knowledge, this will be one of the first studies to look at how patterns of contextually relevant ACEs distinctly cluster in a South African community sample using only child reporting of ACEs. In line with literature that acknowledges ACEs-C may not capture experiences that are disproportionately faced in contexts with higher poverty and crime, we have expanded our conceptualisation of ACEs not only to include some conventionally measured ACEs such as abuse (emotional; physical; sexual) and witnessing domestic violence, but also contextually sensitive ACEs including parental morbidity, parental mortality, transactional sex, bully victimisation, witnessing or experiencing community violence, parental AIDS-affectedness, food insecurity, AIDS-related stigma, and intrahouse discrimination. These ACEs-E were selected based on prior research and expert consultation with local partners during study design, and they reflect adversities previously identified as highly prevalent and developmentally impactful for South African adolescents. Whilst we acknowledge other ACEs such as parental mental illness are commonly included in ACE frameworks; this was not assessed in the child-reported dataset available for this study.

Rather than impose predefined assumptions or expected groupings, we aimed to let the data determine the number and nature of classes. This was particularly appropriate given that, to our knowledge, this was the first study to apply LCA to child-only reported ACEs in a South African context, using both conventional and contextually expanded ACEs. Given the novelty of this approach and the lack of prior LCA research in similar settings, we intentionally did not pre-specify hypotheses about the number or nature of classes.

The two main aims of this study were to (1) use latent class analysis (LCA) to explore the way in which patterns of longitudinally and prospectively measured ACEs form in a South African adolescent community sample, (2) explore the association between a set of demographic covariates (age, gender, location, and poverty) and latent class membership.

## Methods

### Setting

This study is nested in the Young Carer’s Project, a longitudinal sample from the Mpumalanga and Western Cape provinces with data collected over two waves (wave 1 = 2010/2011 and wave 2 = 2011/2012). Two health districts within the two provinces were selected based on having an antenatal HIV prevalence of > 30% and within these districts, stratified random sampling of census enumeration areas was used to determine exact locations for recruitment and data collection.

### Participants

In wave 1, door-to-door sampling was used to recruit 3,515 children aged between 10 and 17 years (56.7% female, mean age = 13.5 years, 50.6% urban location). One child per household was interviewed and random selection of child for interview implemented for households with multiple children. In wave 2, the same children were traced and re-interviewed. The study exhibited a high retention rate between waves 1 and 2 of data collection (retention rate = 96.8%, wave 2 *n* = 3,401). Analysis of how children lost to follow-up differ from children retained at follow-up has already been described in other papers (see Meinck et al., 2017).

### Procedure

All participants and their caregivers provided informed consent prior to taking part in the study. Consent sheets were read aloud by a member of the study team to support instances where literacy was low. Participants were not offered any study participation incentives other than a certificate of participation and refreshments. Participants were free to select the language and location that the interview would be conducted in. The interview was translated from English into local languages isiXhosa, SiSwati, and Xitsonga. Trained interviewers verbally administered the questionnaire at both waves of data collection. The interview lasted approximately 1 h in each wave and the design of the questionnaire mimicked a teen magazine to promote engagement with the interview. Interview staff were local to the areas of data collection and were required to already have experience working with vulnerable children but completed further extensive training prior to the study commencing. Unless interviewers deemed participants to be at significant risk of harm or participants asked for further support from external organisations, confidentiality and privacy were maintained. In cases of significant risk of harm, immediate referrals were completed by the study team.

### Measures

#### ACEs

##### Parental Morbidity

Children had the opportunity to identify multiple sick people in their household via the completion of a Household map. Children were first asked “How many people in the home are sick?” followed by the opportunity to select up to four sick people. If children endorsed their mother or father as being sick at waves 1 and/or 2, they were included as having experienced parental morbidity. Notably, children were given a standardised definition of “sick”, meaning people who are ill over a long period or who must take regular medication.

##### Parental Mortality

Any parental mortality was reported in a Verbal Autopsy Questionnaire (Lopman et al., 2006). Children were scored as having experienced any parental mortality if they endorsed the death of either their mother or father by the end of wave 2.

##### HIV and AIDS-Related Stigma

In both waves, the brief 10-item Stigma-by-Association scale (Mason et al., 2010) was used to measure any HIV and AIDS-related stigma. This scale has been previously validated for use with South African youth (Cronbach’s alpha = 0.89–0.90) (Boyes et al., 2013). The scale captured experiences such as being teased because someone in their family was unwell and other parents not wanting their children to be around the participant. Responses ranged from 0–2 (0 = *not at all*, 1 = *sometimes*, 2 = *all the time*). Across either wave, if participants responded “sometimes” to any of the 10 questions, they were scored as having experienced HIV and AIDS-related stigma.

##### Bullying

In both waves, the 9-item “Social and Health Assessment Peer Victimisation Scale” (SAHA) (Ruchkin et al., 2004) was used to assess bullying victimisation in the past 12 months. The scale has shown excellent reliability in this sample (*α* = 0.81) (Boyes et al., 2014). Verbal bullying victimisation was measured by two items (e.g., “made fun of me for some reason”); physical bullying victimisation was measured by two items (e.g., “hurt me physically in some way”); two items measured bullying victimisation in the form of theft and property damage (e.g., “took something without permission or stole something from me”); relational bullying victimisation was assessed by two items (e.g., “tried to get me in trouble with my friends”); and invasion of physical space was measured by a single item (“made me uncomfortable by standing too close or touching me”). Responses ranged from 1–4 (1 = *not at all*, 2 = *once*, 3 = *2*–*3 times*, 4 = *4 or more times*).

Many bullying definitions recognise bullying as being a repeated experience (Kowalski & Limber, 2013; Olweus, 1993). Subsequently, bullying victimisation was categorised as participants who endorsed four or more victimisation events of any one bullying experience in the past 12 months. Those who experienced less than 4 incidents of single item bullying victimisation in the past 12 months were categorised as not bullied.

##### Family Conflict or Violence

2 items from the UNICEF Measures for National-Level Monitoring of Orphans and Vulnerable Children (Snider & Dawes, 2006) were used to assess past-week family conflict (How many days last week were there arguments with adults shouting at each other) and/or domestic violence (How many days last week were adults hitting each other at home?). Past week frequency of experiences was recorded and ranged from 0 to 7 in total. For binary scoring of family conflict or violence, we implemented a scoring approach that has already been successfully used in a South African sample of children also living in highly deprived areas (Meinck et al., 2015a, b). Participants had to have endorsed at least 1 day of domestic violence experience in the past week (adults hitting each other) and/or at least 3 days of family conflict in the past week (adults shouting or arguing) to count as having experienced living in a home with family conflict or violence.

##### Physical Abuse

The 2 and 3 items used in waves 1 and 2, respectively, were selected from the UNICEF National-level Monitoring of Orphans and Vulnerable Children Measures (Snider & Dawes, 2006), a scale derived from the Parent Child Conflict Tactics Scales (CTSPC) (Straus et al., 1998). Examples of questions included, “How often did caregivers or other adults slap, punch, hit, pinch or pull your ear/hair so that you were hurt or had marks?” and “How often did your caregivers or other adults make you stand or kneel in an uncomfortable position for a long period of time?” Responses were given about past year experiences and ranged from 0–3 (0 = *never*, 1 = *at least once this year*, 2 = *monthly*, 3 = *weekly*). Children were counted as having experienced physical abuse if they responded to any of the experiences happening at least once in that year.

##### Emotional Abuse

Items were also selected from the UNICEF National-level Monitoring of Orphans and Vulnerable Children Measures (Snider & Dawes, 2006). Three items were used in wave 1 and 11 items were used in wave 2. Questions included, “How often did adults say they would invoke ghosts or harm?,” “How often did adults call you dumb, lazy, or other names,” and “How often did adults threaten to leave you and never come back”). Responses were given about past year experiences and ranged from 0–3 (0 = *never*, 1 = *at least once this year*, 2 = *monthly*, 3 = *weekly*). Given repetition is important when conceptualising emotional abuse, weekly or monthly disclosures were counted as 1 in the binary coding and all other responses were counted as 0 (did not experience emotional abuse). The whole physical and emotional abuse scale has previously shown good reliability in the same sample (α = 0.73) (Meinck et al., 2015a, b).

##### Sexual Abuse

South African social workers supported the creation of three custom questions to capture past-year child sexual abuse in wave 1 with binary response options (0 = no, 1 = yes). One item asked about forced sex, one item asked about being touched in a way that felt uncomfortable the last item asked if anyone ever made participants do anything with their private parts or someone else’s private parts that they did not want to do. In addition to the three wave 1 items, wave 2 incorporated three additional past-year items adapted from the Juvenile Victimisation Questionnaire (JVQ) (Finkelhor et al., 2005a, b). An example of the JVQ (Finkelhor et al., 2005a, b) questions that were adapted to suit the South African context included, “How often in the past year has the following happened to you? Someone told you; you look sexy in a way that made you feel uncomfortable?”. South African social workers, children and child protection non-governmental organisations aided modification of wave 2 items. Wave 2 allowed children to specify the chronicity of sexual abuse, with response options ranging from 0–4 (0 = never, 1 = at least once this year, 2 = monthly, 3 = weekly, 4 = happened to me but not in the past year). For creating a binary wave 2 sexual abuse score, children were given a binary sore of 1 (yes) if they had endorsed any lifetime experience of sexual abuse in at least 1 item. Participants got a final binary sexual abuse score of 1 if participants disclosed experiencing any of the sexual abuse items across either wave.

##### Transactional Sex

Over the last decade, “transactional sex” has become an increasingly recognised term in literature, yet research still lacks consensus as to its conceptualisation and implications (Krisch et al., 2019; Williams et al., 2012). The complexity around the term is salient in low-resource contexts, where socioeconomic vulnerability may be a particularly motivational factor for engaging in transactional sex in exchange for economic and material resources (Cluver et al., 2011; Jewkes et al., 2012).

In agreement with prior literature (Doek & Greijer, n.d.); Greenbaum, 2020; Williams et al., 2012) and following the Convention on the Rights of the Child (United Nations, 1989), we are conceptualising children engaging in transactional sex as a form of child sexual abuse and child sexual exploitation. Lifetime exposure to transactional sex was measured using 10 items from the culturally and contextually relevant “National Survey of HIV and Risk Behaviour amongst Young South Africans” (Pettifor et al., 2005). Participants were asked if they had ever received the 10 items in return for sex e.g., money, clothes, school fees, and food (1 = yes, 0 = no). Across either wave, if participants responded “yes” to any of the 10 questions, they were scored as having experienced transactional sex.

##### Intrahouse Discrimination (Favouritism)

Intrahouse discrimination was measured by a single item that asked participants whether the food they received in their household was less, more, or an equal amount compared with other children. This item was developed in the qualitative pilot research. If in either of the two waves participants reported having less food than other children in the household, they were scored as having experienced intrahouse discrimination. Children may have experienced intrahouse discrimination for various reasons including favouritism/childism, or as a form of “deprivational abuse”; “the deliberate or malicious failure to supply the needs of a child” (Golden et al., 2003, p. 105). In this context, favouritism between children in the household is common and often presents as differential treatment across children in the receipt of food or material goods. Furthermore, in this context and in other low-resource, high-deprivation contexts (Boydell et al., 2017), food can be weaponised in households and removed as a form as punishment, thus supporting the validity of this item.

##### Community Violence

Community violence was measured using 4 items adapted from the Child Exposure to Community Violence Checklist (Richters & Martinez, 1993). The items selected represented four of the most experienced community traumas in South Africa based on the 2004/2005 Annual Report of the South African Police Service (SAPS) (SAPS Strategic Management, 2005). The community violence items were dichotomised into two categories; witnessing community violence (seeing someone being shot or stabbed) and directly experiencing community violence (being hit/attacked outside or being robbed).

The experiences measured still represent common community violence issues as seen in the most current SAPS annual report of 2022/2023 (SAPS Strategic Management, 2023). Responses for witnessing shootings, stabbings, and being assaulted were scored as yes (1) or no (0). Robbery was scored using past-year frequency. For the purpose of this paper, if children had witnessed any shooting or stabbings in either wave, they were scored as having witnessed community violence. If children had experienced at least one robbery or any assault in either wave, they were scored as having experienced community violence.

##### Food Insecurity

Items from The National Food Consumption Survey (NFCS) (Labadarios et al., 2005) were used to assess past-week food insecurity. In line with the cut-off for food insecurity seen in prior publications using the same dataset (Cluver et al., 2016), we scored children as having experienced food insecurity if they reported at least 2 days in the past week without enough food in either wave of data collection. Whilst the NFCS-based items lack detailed psychometric validation, their development and use in South African populations support their validity as indicators for food insecurity (Misselhorn & Hendricks, 2017).

We want to address why the decision was made to include food insecurity as an ACE but have poverty remain as a covariate. Food insecurity has been associated with significant long-term cognitive, socio-emotional, and behavioural outcomes, and this finding is also independent of poverty or socioeconomic background (de Oliveira et al., 2020; Perez-Escamilla & de Toledo Vianna, 2012; Shankar et al., 2017), which supports its inclusion as an ACE.

##### Parent(s) Affected by AIDS

We created a parent(s) affected by AIDS variable in which children would get a binary score of 1 if they had reported parental sickness from AIDS or parental death from AIDS in either or both waves of the study. In both waves*,* Verbal Autopsy methods (Lopman et al., 2006) were used to obtain information about whether the children had lost any or both parents to AIDS and whether the children had any parent sick with AIDS. The verbal autopsies were initially administered and tested in Zimbabwe but have since been successfully applied and validated in a South African context showing high sensitivity (89%) and specificity (93%) for adult infectious diseases such as AIDS (Kahn et al., 2000). In addition, despite verbal autopsies often being confined to adult reporting and/or self-reporting, findings have shown children can reliably report on adult’s provisional HIV status (Becker et al., 2015). If children endorsed single or dual parental death by AIDS by the end of wave 2 and/or endorsed at least one parent with an AIDS-related illness by the end of wave 2, they were scored as having a parent(s) affected by AIDS.

#### Covariates

The socio-demographic variables location (rural vs. urban), province (Western Cape vs. Mpumalanga), age, gender, and poverty were included as covariates.

##### Age

The mean age at wave 2 was selected as the age covariate. To help meaningful interpretation, we centred the age around the mean before adding it into our latent class model.

##### Gender and Urban Versus Rural

Gender was captured as boy or girl and the location measure of urban or rural was based on what field site the participant resided. There were slight discrepancies between wave 1 and wave 2 gender and location and thus we chose to use wave 2 data as it was the most up to date data.

##### Province

 The province in which children resided was included as a binary variable (Western Cape = 0 and Mpumalanga = 1). Province (Mpumalanga vs. Western Cape) was included as a covariate to explore whether patterns of ACE exposure varied by structural and geographic context. Mpumalanga and the Western Cape represent distinct structural settings. Mpumalanga was more rural, with higher rates of poverty and more limited access to health and social services, whilst the Western Cape had stronger infrastructure and service delivery systems (Hall, 2012; Statistics South Africa, 2021). As such, province was included as a proxy for underlying contextual and structural variation that may influence children’s exposure to and reporting of ACEs.

##### Household Poverty

Poverty was measured by assessing access to 8 of the most socially perceived basic needs for all children and adolescents in South Africa i.e., being able to afford 3 meals, toiletries, school uniform, school fees, clothes, medical care, and two pairs of shoes (Barnes & Wright, 2012). The scale has previously been used in the South African Social Attitudes Survey (Pillay et al., 2006). A score of 1 indicated the participants could afford the item. For our analysis, we reverse coded the scores (1 = cannot afford the item, 0 = can afford the item) and created a total poverty score out of 8 for each participant based on the sum of items they could not afford. In this context, we are viewing poverty as a structural driver of ACEs (n.b., the term ‘ACEs’ including food insecurity) that is governed by complex economic, political, and cultural factors. Despite research demonstrating a strong relationship between childhood poverty and an increased risk of experiencing ACEs (Walsh et al., 2019), it is important to acknowledge that children from any socioeconomic background can experience ACEs.

Wave 2 poverty was used as it contained the most up to date data. The poverty index we used (Cronbach’s alpha = 0.81) (Barnes & Wright, 2012) had an item that measured receiving three meals a day. We chose to remove this item from the index to ensure our poverty and food insecurity measures were capturing two distinct constructs. The alpha value of the poverty index remained adequate upon removal of the food insecurity item (Cronbach’s alpha = 0.81).

### Statistical Analysis

Latent class analysis (LCA) was conducted using Mplus (version 8.9) (Muthén & Muthén, 2017). LCA is a subset of finite mixture modelling that assists in the identification of at least two unobserved heterogeneous subgroups in a population (Lanza et al., 2013; Masyn, 2013). All ACE variables were included in the LCA models to permit the extraction of latent classes that would indicate patterns of childhood adversity. Maximum likelihood methods were used to estimate competing unconditional latent class models. One hundred random sets of starting values along with 50 optimisations were generated, and then, 50 iterations were specified. We ran ten separate LCA analyses specifying 1–10 classes.

The most appropriate number of classes and thus the best model fit was decided using multiple statistical criteria including the Log Likelihood (Morgan, 2015), the Akaike information criterion (AIC) (Akaike, 1987), the Bayesian information criterion (BIC) (Schwarz, 1978), and the sample-size adjusted Bayesian information criterion (SABIC) (Sclove, 1987), where smaller values were indicative of better model fit. We also examined the *p*-value for the Vuong-Lo-Mendell-Rubin adjusted likelihood ratio test statistic (VLMR) (Lo et al., 2001; Vuong, 1989) and as recommended and followed in literature (Masyn, 2013; Weller et al., 2020), we checked that all classes in each model included at least 5% of the total sample size. Lastly, we assessed the entropy of the latent class models which ranged from 0 to 1. Entropy was not used to aid the selection of the optimal class solution in line with recommendations (Masyn, 2013); rather it was used to assess the goodness of fit of cases into classes. Higher entropy values indicate better separation of classes (Sinha et al., 2021) and whilst there is no clear cut-off used for entropy, values above 0.8 have been deemed acceptable (Weller et al., 2020). Variable-specific entropy contribution (univariate entropy) was also examined to assess the extent to which each indicator informs the identification of latent classes (Asparouhov & Muthén, 2018).

Upon selection of the optimal class solution, we ran an automatic three-step model using the R3STEP auxiliary command to examine the relationship between class membership and the covariates (Asparouhov & Muthén, 2013; Morin et al., 2020; Vermunt, 2010). Using the R3SETP command, the covariates (gender, age, location, province, and poverty) are treated as latent class predictors and minimises the effects of the covariates on class formation. Literature has acknowledged the criteria used to determine optimal class solution in latent class analyses is often changing and lacks standardisation of implementation and unanimity of agreement (Weller et al., 2020). We kept an openness to discuss and review the final class model. We used a combination of statistical and theoretical criteria to guide model selection, as described below.

Model fit was assessed for 2-class through to 10-class models. As shown in Table 1, the VLMR became non-significant at alpha = 0.05 at the 5-class solution, indicating the 4-class solution was the optimal model. However, the Log Likelihood, AIC, BIC, SABIC, and BLRT all indicated we should proceed (by varying degree) beyond the 4-class model. A lack of consensus for the optimal model selection indicted by an absence of convergence across indices led authors to plot the BIC values (see Supplementary Information) to determine what model began to yield “diminishing returns” in value for each added class – an approach that has been deemed acceptable practice in prior literature (Nylund-Gibson & Choi, 2018). The BIC plot revealed diminishing returns at the 5-class solution with only a marginal decrease between the BIC value for the 5 (53169.266) and 6-class (53136.218) models.

**Table 1:** Fit statistics for latent class models estimating 2–10 latent classes (*n* = 3401)

*K*	LL	AIC	BIC	SABIC	VLMR	VLMR *p*-value	Entropy	BLRT	BLRT *p*-value	Smallest class size count (%)
2	− 26,827.778	53,713.556	53,891.379	53,799.232	1892.864	<.0001	0.627	− 27,774.21	<.0001	1295 (38%)
3	− 26,511.053	53,110.107	53,379.907	53,240.098	633.449	<.0001	0.757	− 26,827.778	<.0001	975 (28.7%)
4	− 26,396.543	52,911.086	53,272.863	53,085.393	229.021	<.0001	0.771	− 26,511.053	<.0001	582 (17.1%)
5	− 26,283.755	52,715.511	53,169.266	52,934.133	225.575	0.0535	0.661	− 26,396.543	<.0001	477 (14%)
6	− 26,206.243	52,590.486	53,136.218	52,853.424	155.025	0.2502	0.657	− 26,283.755	<.0001	340 (10%)
7	− 26,143.789	52,495.578	53,133.288	52,802.831	124.908	0.1668	0.65	− 26,206.243	<.0001	315 (9.3%)
8	− 26,099.035	52,436.071	53,165.758	52,787.639	89.507	0.6173	0.655	− 26,143.789	<.0001	282 (8.3%)
9	− 26,061.308	52,390.616	53,212.281	52,786.5	75.454	0.3017	0.658	− 26,099.035	<.0001	211 (6.2%)
10	− 26,029.724	52,357.449	53,271.09	52,797.648	63.168	0.225	0.697	− 26,061.308	<.0001	187 (5.5%)

*LL*, log likelihood; *AIC*, Akaike information criterion; *BIC*, Bayesian information criterion; *SABIC*, Sample-size adjusted BIC; *VLMR*, Vuong-Lo-Mendel Rubin (*p*-value); *BLRT*, bootstrap likelihood ratio test (*p*-value)

After assessing all statistical criteria and before selecting an optimal solution, we explored what class model had most theoretical interpretability. We drew on ecological and developmental theories, which suggest that childhood adversities cluster in ways that reflect broader structural and relational vulnerabilities within families and communities. The 5-class model offered the most nuanced and context-sensitive class structure, with clearly distinguishable classes that aligned not only with statistical indicators and parsimony but also with our deep familiarity of the South African context—including prevalent adversities such as community violence, parental death, and intersecting household vulnerabilities.

## Results

### Sample Characteristics

There were 3,401 adolescents in the sample. Descriptive statistics for the sample are presented in Table 2, which includes the demographic variables gender, age, poverty levels, location setting, ACEs in both waves and an ACE count which was created using data from both waves.

**Table 2:** Descriptives of ACE prevalence and sociodemographic data for entire sample, with *n* (%) presented split by wave and *n* (%) presented combined across waves using the scoring outlined in this paper

Sociodemographics	Wave 1 *n* (%)	Wave 2 *n* (%)	
Gender			
Boy	1475 (43.4)	1482 (43.6)	
Girl	1926 (56.6)	1919 (56.4)	
Mean age	13.4 years	14.7 years	
Province			
Western Cape	1753 (51.5)	1753 (51.5)	
Mpumalanga	1648 (48.5)	1648 (48.5)	
Location			
Rural	1681(49.4)	1692(49.8)	
Urban	1720(50.6)	1709(50.3)	
Poverty (*n* necessities unable to afford)			
0	723 (21.3)	695 (20.4)	
1	696 (20.5)	729 (21.4)	
2	439 (12.9)	382 (11.2)	
3	349 (10.3)	334 (9.8)	
4	322 (9.5)	334 (9.8)	
5	313 (9.2)	329 (9.7)	
6	317 (9.3)	318 (9.4)	
7	182 (5.4)	228 (6.7)	
8	59 (1.7)	45 (1.3)	
ACEs (combined across both waves)	Wave 1 *n* (%)	Wave 2 *n* (%)	Combined *n* (%) (either endorsed in wave 1 or 2)
AIDS-related stigma	1107 (32.6)	728 (21.4)	1472 (43.3)
Parental death	930 (27.3)	895 (26.3)	1120 (32.9)
Parental morbidity	1000 (29.4)	619 (18.2)	1237 (36.4)
Parent(s) affected by AIDS	1318 (38.8)	986 (29)	1684 (49.5)
Parental AIDS-sickness	1063 (31.3)	602 (17.7)	1360 (40)
Parental death by AIDS	440 (12.9)	507 (14.9)	640 (18.8)
Bullying	1023 (30.1)	776 (22.8)	1486 (43.7)
Intrahouse Discrimination	147 (4.3)	113 (3.3)	250 (7.4)
Domestic Violence/household conflict	288 (8.5)	220 (6.5)	465 (13.7)
Sex abuse	407 (12)	673 (19.8)	930 (27.3)
Transactional sex	102(3)	140(4.1)	217(6.4)
Emotional abuse	654 (19.2)	707 (20.79)	1165 (34.3)
Physical abuse	1365 (40.1)	1292 (38)	2046 (60.2)
Community violence			
Witness community violence	1406 (41.3)	1338 (39.3)	1906 (56)
Experience community violence	1523 (44.8)	1312 (38.6)	2132 (62.7)
Food insecurity	828(24.4)	830 (24.4)	1323(38.9)
ACE count		Combined *n* (%) (either endorsed in wave 1 or 2)	
0		51 (1.5)	
1		167 (4.9)	
2		291 (8.6)	
3		443 (13)	
4		526 (15.5)	
5		484 (14.2)	
6		437 (12.9)	
7		378 (11.1)	
8		290 (8.5)	
9		173 (5.1)	
10		107 (3.2)	
11		38 (1.1)	
12		12 (0.4)	
13		2 (0.1)	
14		2 (0.1)	

On average across the first two waves, rates of poverty were high with children lacking almost 3 (*n* = 2.7) of the 8 necessities for all children and teenagers in South Africa as identified by the South African Social Attitudes Survey (Pillay et al., 2006). Combining data from waves 1 and 2 showed 39% of children (*n* = 1323) had experienced food insecurity. By the end of wave 2 data collection, 56% of the 3,401 retained children had witnessed community violence including someone being shot or stabbed (*n* = 1906) and 63% of children had experienced community violence through either being hit or attacked outside or robbed (*n* = 2132).

**Fig. 1:**
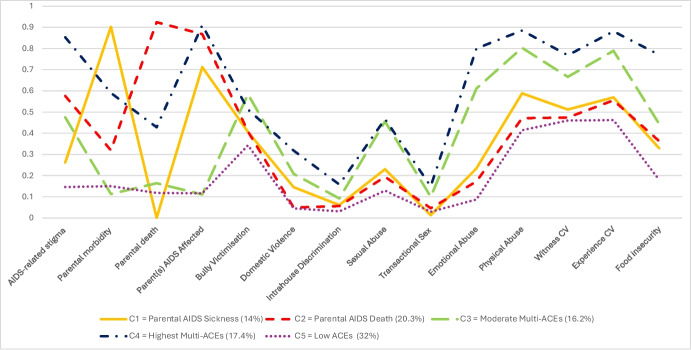
The 5-class model of ACEs and response probabilities for the 14 ACEs for each class. Note. “CV”, Community Violence; class 1, Highest Multi-Type ACEs; class 2, High Parental AIDS-Affectedness and Parental Death; class 3, High Parental AIDS-Affectedness and Parental Morbidity; class 4, Moderate Multi-Type Abuse; class 5, Low ACEs

The 5-class solution Class characteristics can be seen in Fig. 1 and the 4-class and 6 -class solutions can be found in Supplementary Information. Bullying victimisation, transactional sex, domestic violence, witnessing community violence and intrahouse discrimination all had univariate entropy of < 0.05. Sensitivity analysis showed removing these variables still produced conflicting fit indices, only slightly increased entropy and yielded similar class probabilities and characterisations for the ACEs that remained in the model. Bullying, domestic violence, witnessing community violence, and transactional sex have been widely recognised and researched as ACEs in literature and thus whilst not necessarily contributing significantly to class structure or differentiation, we believed deserved to remain in the model theoretically. In a low-resource context with high levels of poverty, intrahouse discrimination was a context-specific variable used to capture differential treatment of children within the house instead of a more traditionally conceptualised way of measuring neglect. Again, we believed this gave a strong enough precedent for the variable to remain in the model despite univariate entropy of < 0.05.

To differentiate probabilities of each ACE within each class, we defined the probability of ACEs as low (< 0.41), moderate (0.41–0.66), and high (> 0.66). First, classes were categorised as having low, moderate, or high levels of adverse childhood experiences. Classes with high levels of ACEs were those that had at least four ACEs with high probabilities; moderate ACE classes were those that had at least four ACEs with moderate or high probabilities; and low ACE classes were those that had less than four ACEs with moderate or high probability. The 5-class model produced one class with high levels of ACEs (class 1), one class with low levels of ACEs (class 5) and 3 classes with moderate levels of ACEs (classes 2, 3, and 4).

### Class 1 (Highest Multi-Type ACEs) and Class 4 (Moderate Multi-Type Abuse): The Compounding Impact of Parental AIDS Affectedness

Whilst being in two different probability categories of moderate (class 3) and high (class 4) levels of ACEs, these classes served as a unique opportunity to compare children who experienced the same pattern of ACEs and how the probability of experiencing these ACEs increased in the class where participants had a parent affected by AIDS. First, compared to all other classes, class 4 (highest ACEs) had the highest probability of experiencing over 78% (*n* = 11) of included ACEs. This excluded parental sickness (0.59) and death (0.428), where the highest probabilities were seen in classes 1 and 2 in which these ACEs served as core characterisations and excluded bully victimisation (0.512), where the highest probability was seen in class 3 (moderate multi-type violence). In contrast to class 4’s moderate probabilities of parental morbidity and mortality, class 3 had lower probabilities of 0.113 and 0.164, respectively. Despite the fact class 3 and 4 both presented with low probabilities of transactional sex (0.1–0.149), domestic violence (0.208–0.318), and intrahouse discrimination (0.092–0.158), they were still the classes that exhibited the highest relative likelihoods of these ACEs with class 4 (highest ACEs) presenting with the highest likelihoods for these ACEs across all classes. Participants in class 3 and class 4 had almost similar moderate probabilities of experiencing sexual abuse (0.4656–0.467) and both classes had moderate probabilities of experiencing bully victimisation (0.512–0.584). They also had similarly high probabilities of witnessing (0.666–0.768) and experiencing (0.789–0.881) community violence, along with high probabilities of experiencing physical abuse (0.803–0.885).

We already discussed that classes 3 and 4 were distinct in their likelihoods of experiencing parental sickness and death, but class 3 and class 4 became further distinguishable from each other when looking at the likelihood of having at least one parent that was AIDS-affected. Class 4 had very high probability of experiencing a parent that was AIDS-affected (0.91), whereas class 3 had a low probability (0.111) of having AIDS-affected parent(s). There was also a difference in the probability of experiencing AIDS-related stigma, with the participants in class 4 having a high probability of AIDS-related stigma (0.853) and those in class 3 having a moderate likelihood of AIDS-related stigma (0.475). The final distinction between these classes was seen in emotional abuse and food insecurity, where class 4 presented high likelihoods of these experiences (0.8 and 0.77, respectively) compared to the moderate probabilities seen in class 3 for food insecurity (0.444) and emotional abuse (0.611).

### Class 2 (High Parental AIDS-Affectedness and High Parental Death) and Class 3 (High Parental AIDS-Affectedness and High Parental Morbidity): Moderate ACE Exposure

Classes 1 and 2 both had moderate patterns of ACEs. These classes had low likelihoods of experiencing abuse (sexual: 0.194–0.23; emotional: 0.172–0.234), food insecurity (0.329–0.363), intrahouse discrimination (0.056–0.061), bully victimisation (0.406–0.408), domestic violence (0.049–0.145), and transactional sex (0.013–0.046). Both classes had similar, moderate likelihoods of physical abuse (0.471–0.588) and both witnessing (0.474–0.512) and experiencing (0.555–0.569) community violence, as also seen in the low-level ACE class (Class 5). When looking at the adverse childhood experiences that have high probabilities in these classes, we can see both classes had high likelihoods of having at least one parent that is affected by AIDS (0.712–0.868). However, a clear distinction in experiences emerged between classes 1 and 2 when looking at parental morbidity and mortality. Class 2 was characterised by a high likelihood of parental death (0.924), a low likelihood of parental sickness (0.319), and class 1 showed the opposite pattern with no likelihood of parental death (0.0) and a high likelihood of parental sickness (0.902). Another distinction between the participants in classes 1 and 2 was that participants in class 2 who experienced a high likelihood of parental death (likely parents that had been affected by AIDS given the high probability for this ACE) showed a moderate likelihood of experiencing AIDS-related stigma (0.576). In contrast, children in class 1 who had a high probability of parental sickness (likely also affected by AIDS) did not exhibit the same moderate likelihood of AIDS-related stigma (0.262**).**

### Class 5: Low ACE Exposure

Class 5 was the only class to exhibit a low likelihood of ACEs. Out of the five classes, this class had participants with the lowest likelihood of over 70% of the ACE categories, including experiencing abuse (sexual: 0.129; physical: 0.414; emotional: 0.087), experiencing AIDS-related stigma (0.146), food insecurity (0.18), intrahouse discrimination (0.031), domestic violence (0.045), and bullying (0.343). Though having the lowest likelihood of exposure to community violence, the participants in this class still had a moderate likelihood of both witnessing (0.46) and experiencing (0.462) violence in their communities. In addition, despite not showing the lowest probability out of all classes for experiencing parental morbidity (0.15), parental death (0.118), parent(s) being AIDS affected (0.116) and transactional sex (0.029), participants in this class still had low likelihoods of experiencing these ACEs.

### Covariates

Table 3 presents results for four multinomial logistic regression analyses with class 5 (Low ACEs) as the reference class. In each analysis, classes were regressed on the five covariates poverty, gender, age, province, and location. The results from Table 3 suggest there was no significant difference in location (rural vs peri-urban) between each class and the low ACE class. However, age, gender, poverty, and province all showed significant differences across classes. Firstly, there were clear gender differences between classes 3 (moderate, multi-type violence ACE class), class 4 (highest ACE class), and the low ACE class, with girls being significantly more likely to be in class 3 and 4 where the highest probabilities of ACEs were observed. In addition, compared to the low ACE class, classes 1–4 had significantly higher levels of poverty with the highest ACE class (class 4) showing the highest levels of poverty. Excluding class 1 (High Parental AIDS-Affectedness and High Parental Morbidity), all other classes were significantly more likely to have older adolescents compared to the low ACE reference class, with the oldest ages seen in classes 3 and 4. Lastly, compared to the low ACE class, class 2 (High Parental AIDS-Affectedness and High Parental Death) was significantly more likely to have children from the Mpumalanga province.

**Table 3:** Regression analyses results for the association between class membership and the covariates poverty, gender, age, location, and province

Class	Covariate	Estimate (*β*)	SE	*p*-value
1 (High Parental AIDS-Affectedness and High Parental Morbidity)	Poverty	0.176	0.052	0.001
	Age	0.05	0.039	0.195
	Gender	0.146	0.164	0.373
	Location	0.108	0.167	0.518
	Province	0.327	0.195	0.095
2 (High Parental AIDS-Affectedness and High Parental Death)	Poverty	0.235	0.047	< 0.0001
	Age	0.157	0.031	< 0.0001
	Gender	0.229	0.131	0.079
	Location	− 0.171	0.137	0.214
	Province	0.959	0.178	< 0.0001
3 (Moderate Multi-Type Violence)	Poverty	0.256	0.058	< 0.0001
	Age	0.181	0.042	< 0.0001
	Gender	0.4	0.17	0.019
	Location	− 0.15	0.175	0.39
	Province	− 0.254	0.211	0.228
4 (Highest Multi-Type ACEs)	Poverty	0.579	0.051	< 0.0001
	Age	0.18	0.034	< 0.0001
	Gender	0.536	0.146	< 0.0001
	Location	0.035	0.152	0.818
	Province	0.213	0.198	0.283

Class 5 (Low ACEs) was used as the reference group for each covariate analysis

## Discussion

Implementing a person-centred approach such as LCA to explore patterns of ACEs in a South African sample of adolescents living in high deprivation allowed us to gain an insight into the dynamic, intricate, and non-linear way ACEs co-occur (Masyn, 2013). Our study identified five distinct classes of ACEs with 1 low probability ACE class, 3 moderate probability ACE classes and 1 high probability ACE class. Our findings align with prior LCA work in African community settings that suggests children and adolescents experience diverse patterns of adversity and those from the same or similar settings should not be viewed as homogeneous groups with the same ACEs (Ferrajāo et al., 2023; Miedema et al., 2024; Zietz et al., 2020).

Participants in the low probability class 5, named low ACE exposure, had a low likelihood of experiencing any family/household adversity and most forms of violence exposure, yet still had a moderate probability of experiencing (being assaulted or robbed) and witnessing (seeing a stabbing or shooting) community violence. Using Finkelhor and colleagues (2009) polyvictimisation theory as a lens to interpret this finding, this result suggests that despite avoiding many forms of adversity, these adolescents may reside in particularly violent or dangerous communities where the risk of witnessing or experiencing violence is high, but they do not seem to have as many risk pathways that would result in experiencing multi-ACE exposure such as living in a particularly violent family home or having a chaotic family environment. In addition, despite being the lowest probability of physical abuse relative to the other classes, the probability of experiencing physical abuse was still moderate, suggesting even in the lowest ACE class there is still a risk of household-level violence. With over 60% of the adolescent sample experiencing physical abuse, it is not particularly surprising that children in this lowest ACE class still have a moderate probability of experiencing this form of adversity. Looking at this finding through the lens of an ecological framework (Bronfenbrenner, 1979) emphasises the importance of considering the multi-layered contexts and ecosystems in which children exist in, including acknowledging risk factors outside of the individual and household level.

Despite there being three classes with moderate ACE exposure (defined by classes that had 4 or more ACEs with a probability of 0.41–0.6), we have chosen to discuss two of these alongside each other (classes 1 and 2) and leave class 3 to be discussed with class 4, the highest ACE class. Class 1 was named “High Parental AIDS-affectedness and Parental Sickness” since the defining ACEs of this class were having a sick parent and parental AIDS-affectedness. Class 2 was named “High Parental AIDS-affectedness and Parental Death”, as it was distinct from the other classes due to the high likelihood of participants having a parent that had died and been affected by AIDS. Whilst we are unable to disentangle the causes of death and sickness for parents in the parental morbidity and parental death classes, the co-occurring high probabilities of having an AIDS-affected parent (a parent that is sick with AIDS or has died from AIDS) in both classes may indicate that most of the participants in the high parental death class have had parents that have died from AIDS and most participants in the high parental sickness class have experienced a parent sick with AIDS. Community violence remained a moderate likelihood in these classes as seen in the low ACE class, potentially again a result of residing in violent communities. Notably, class 2 which had high parental death and AIDS-affectedness was also characterised by participants experiencing AIDS-related stigma.

Research has reported that children orphaned by AIDS not only experience more stigma than children who do not have a family affected by AIDS-related death (Chi & Li, 2013; Nyamukapa et al., 2010) but also suggests stigma as a significant risk factor for psychopathology in AIDS orphans (Cluver et al., 2008). Whilst literature also acknowledges children may experience stigma by association with a parent sick with AIDS (Bogart et al., 2008), our findings may suggest that children who have been orphaned by AIDS are more likely to experience AIDS-stigma than those who have experienced parental AIDS-sickness. Whilst speculative, this may have been due to the comparatively greater severity of the experience of parental death versus sickness. Research highlights multiple losses that are associated with AIDS orphanhood, including not only the loss of what could be a loving relationship with their parent(s), but the loss of social capital, money and education (Harms et al., 2010). Furthermore, children who have parents die from AIDS often go to live with new caregivers where they are stigmatised and treated unfairly compared to the other children in the household (Harms et al., 2010; Strode & Grant, 2001).

Perhaps the most interesting finding in our LCA results was looking at classes 3 and 4 alongside each other as a case study into the compounding effect of having an AIDS-affected parent on the likelihood of multi-ACE exposure. Class 4 was labelled the “highest multi-type ACEs” and class 3 was labelled “moderate multi-type violence”. As discussed, both classes had the same core cluster of ACEs, including sexual abuse, emotional abuse, physical abuse, bully victimisation, community violence, stigma, and food insecurity. However, the classes became distinct when looking at parental sickness and death. Whilst class 3, “moderate multi-type violence”, avoided any parental AIDS-affectedness, including parental sickness and death, participants in class 4 (the highest ACE class) exhibited very high probability of parental AIDS-affectedness, possibly explaining this subgroup’s subsequent high likelihood of AIDS-related stigma and moderate likelihoods of parental sickness and death. Furthermore, in class 4, we saw that a compounding effect on ACE exposure arose once children had a parent affected by AIDS; the probabilities of experiencing the same pattern of ACEs as seen in class 4 mostly increased in comparison, with a particular increase in the burden of food insecurity, physical abuse, and emotional abuse. In line with prior findings from South Africa, we suggest that the reason for this increase in likelihood of these ACEs is due to the fact children in AIDS-affected families—whether impacted through illness or death—are at a significantly increased risk of child abuse victimisation (Cluver et al., 2011; Meinck et al., 2015a, b). For example, Cluver and colleagues (2011) found adolescents affected by AIDS orphanhood and parental AIDS sickness were up to three times more likely to experience severe emotional and physical abuse than those living in households not affected by AIDS.

Meinck and colleagues (2015) found extreme poverty was also a risk factor for child abuse in families affected by AIDS-illness. Class 1, where we saw the highest level of parental AIDS-affectedness and child abuse, was also the class that had the highest levels of poverty and the highest likelihood of experiencing food insecurity. This not only aligned with Meinck et al. and’s (2015a, b) prior findings, but a wealth of additional literature citing strong associations between poverty, food insecurity, and child maltreatment (Hunter & Flores, 2021; Kim et al., 2022).

Despite the low probability of any parental AIDS-affectedness or parental sickness/death, class 4 still had a moderate likelihood of AIDS-related stigma. We propose this moderate likelihood exists because the questions pertaining to AIDS-related stigma asked about any household member that was sick or had died and were not exclusive to the parents. Literature acknowledges the stigma children may feel when residing with an AIDS-sick family member (Surkan et al., 2010). This class thus may be unique in that the burden of AIDS-stigma was arising from another household member being affected by AIDS and not their parents.

Classes 3 and 4 were also the only classes where adolescents exhibited a moderate probability of bully victimisation. A recent systematic review spanning two decades of research (Merrin et al., 2024) found significant positive associations between cumulative adversity, child maltreatment, family violence, physical abuse, and bully victimisation. As classes 3 and 4 were the two subgroups in which adolescents had the highest likelihood of exposure to multiple forms of violence/abuse, our findings align with extant literature.

Important sociodemographic differences (gender, age, poverty, and province) seemed to occur with class membership, suggesting a probability of group differences across classes beyond the reporting of different ACEs. We saw adolescent girls were more likely to be in the classes with higher levels of adversity compared to the lower ACE class and were particularly more likely to be in the two classes (3 and 4) where there was a moderate likelihood of experiencing sexual abuse compared to the lowest ACE class. This finding aligns with most literature citing girls are more likely to experience childhood sexual abuse than boys (Dierkhising et al., 2019; Dube et al., 2005; Finkelhor, 1994).

Poverty levels were also significantly higher in classes 1–4 compared to the low ACE class. This finding was expected as aforementioned, there is a consensus in literature that children living in poverty have a disproportionately higher risk of experiencing ACEs (Walsh et al., 2019). Furthermore, the highest poverty levels were seen in class 4 which displayed the highest levels of food insecurity and other childhood adversities. This finding builds on prior research that has found strong associations between poverty, food insecurity, and ACEs (Jackson et al., 2019; Wight et al., 2014). Compared to the low ACE class, classes 2 (high parental death class), 3 (moderate multi-type violence class) and 4 (highest ACE class), had significantly older adolescents. The oldest children in the sample were seen in classes 3 and 4—the only classes where sexual abuse was moderately likely. Past literature has cited significant age-related differences in exposure to sexual abuse (Ajduković et al., 2013; Finkelhor et al., 2013), with older children having greater risk of exposure compared to younger children. However, there could also be a more general cumulative risk of ACE exposure increasing as time lapses.

Compared to the low ACE class, class 2 (high parental AIDS-affectedness and high parental death) was significantly more likely to have children from the Mpumalanga province than the Western Cape province. Whilst the underlying reasons for this difference remain unclear it may reflect broader contextual differences between provinces during the time of data collection, including variation in household composition, caregiving arrangements, or healthcare access. Furthermore, HIV prevalence in the two sampled districts was similarly high at the time of wave 1 recruitment (> 30%), but provincial antenatal HIV prevalence data later showed a sharper decline in the Western Cape by 2012 (Bor et al., 2013; Shisana et al., 2014). Although HIV prevalence often remain stable in general populations receiving ART, declines in antenatal HIV prevalence can reflect successful public health interventions, including reduced incidence among young women, earlier treatment, or changing fertility patterns. Nevertheless, given the complexity of interpreting these trends and that linking these class differences directly to provincial HIV prevalence trends is speculative, we have therefore chosen not to draw conclusions on this basis.

A growing body of research has shown the value of person-centred approaches to explore ACEs (Ferrajão et al., 2022; Negriff, 2021; Shin et al., 2018) and has also found class membership routinely predicts poor health outcomes with varying risk (Lanier et al., 2018; Niño et al., 2023; Parnes & Schwartz, 2022). This study supports considering the multiplicative, not additive, effects of ACEs.

### Research Implications

The current results suggest ACEs co-occur in unique patterns and recommends future research continues with the use of person-centred approaches to ACEs that move beyond looking at single ACE effects to inform ACE prevention strategies. The results also highlight that experiences broadly regarded as more traumatic events such as community violence often co-occur with experiences viewed as less severe events such as parental morbidity which highlights the complex nature in which ACEs co-occur. Gaining further understanding into how ACEs may cluster together is a pivotal first step into understanding specific risk profiles of adversity for later-life poor outcomes (Wang et al., 2022). As ACEs are increasingly screened for in healthcare settings, it is vital to begin to understand how interventions and treatment options may differ in effectiveness for patients that present with specific patterns and combinations of ACEs.

The results also highlight the importance of expanding the conceptualisation of ACEs-C for highly deprived contexts with samples that differ from the white, educated, middle-class demographics that were seen in the pioneering ACE studies (e.g., Felitti et al., 1998). The current results show in this sample of Black South African adolescents living in highly deprived settings, ACEs-E are more important than ACEs-C in distinguishing latent classes. Furthermore, the most reported ACEs were parental AIDS-affectedness, parental mortality, parental morbidity, community violence and food insecurity – all experiences that were included with context sensitivity in mind and would not have been captured if not for expanding our conceptualisation of ACEs.

We also emphasise the present-day relevance of HIV and AIDS-related adversity. Data suggests this remains a critical and contemporary global issue for children, with an estimated 630,000 HIV-related deaths in 2024 (World Health Organization, 2025). The ongoing HIV epidemic remains salient in low-resource communities where access to healthcare and social support is limited (Barr et al., 2021).

Whilst the scale-up of antiretroviral therapy (ART) has significantly improved treatment outcomes and life expectancy (Michael et al., 2021), we still need to recognise that historical HIV and AIDS-related adversity has had enduring consequences for a generation of children and young people who grew up during the epidemic when ART was largely inaccessible. For example, many children—including those in the original W1 sample—grew up in households where they lost one or both caregivers to AIDS-related illness, often in environments marked by secrecy, stigma and inadequate social support (Cluver & Gardner, 2007; Cluver et al., 2011). Consequently, although HIV/AIDS-related illness and death have decreased, the effects of these past adversities persist, impacting mental health outcomes, social functioning and economic stability (Li et al., 2009; Tutlam et al., 2023).

Understanding the longer-term impact of HIV and AIDS-related adversity in childhood is critical to assess the current burden of mental illness and other adverse outcomes among affected generations today. Policymakers should therefore prioritise addressing the lasting effects of the epidemic on children and families, ensuring mental health interventions and social protection programs acknowledge the needs of these generations to mitigate its intergenerational impacts and the risk for additional adversity in childhood and across the life course.

In addition, these findings reflect childhood experiences during 2010–2012, when antiretroviral treatment (ART) access was not yet universal. Since then, The United States President’s Emergency Plan for AIDS Relief (PEPFAR) played an important role in ART rollout whilst providing care and support for over 7.1 million orphans and vulnerable children and their caregivers (Cluver et al., 2023; U.S. Department of State, 2022). However, with PEPFAR under threat and persistent gaps in ART coverage, similar patterns of HIV-and AIDS-related adversities could re-emerge unless the South African government strengthens its response efforts or US Congress renews funding commitments.

### Practice Implications

To explore how our findings may align with practice realities, we held informal discussions with three South African social workers from the third wave of this study (Meinck et al., 2023); two who currently practice within Mpumalanga province and one who previously did. All three have experience working in these community prior to the widespread rollout of ART. The discussions summarised below offer anecdotal perspectives that, whilst not generalisable, illustrate how our findings may resonate with real-life, present-day casework.

The social workers indicated that the results of our study reflected the types of cases they routinely manage; particularly the high prevalence and co-occurrence of ACEs in complex patterns. They described how caseloads in these low-resource communities often require highly skilled responses and careful prioritisation. They also noted a systemic lack of funding to provide appropriate resources such as emotional support and training to manage such complexity effectively. In discussing families affected by AIDS, they reflected that it is now much less common in their practice to associate the term “HIV” with “AIDS”, attributing this to the widespread availability of antiretroviral therapy (ART) (Burger et al., 2022). As ART has improved survival and health outcomes (World Health Organization, 2016), the term “AIDS” is used less frequently, and the experience of AIDS-related sickness or death appears less visible in daily cases.

However, in line with the study findings, they noted HIV-related stigma persists, particularly where the children have lost a parent and now reside with non-parental family members or foster carers. They described how these children may continue to face further childhood adversity such as food insecurity or abuse, and highlighted the importance of the foster child grant as a protective factor. Their experiences suggested the grant may incentivise more supportive and appropriate care from new caregivers.

These reflections align with the socio-ecological pathways identified in our study and highlight the value of a whole-family approach in social work practice, especially in households affected by HIV and AIDS, This paper provides necessary impetus for better standardisation within social work practice of additional resources such as further training and emotional support to better equip social workers who encounter and manage such complex caseloads daily. Nonetheless, we emphasise these practitioner insights are illustrative rather than conclusive and we remain tentative in extrapolating their observations to current patterns or national trends.

### Limitations

The main advantages of using prospective reporting of ACEs included allowing for covariates to be measured at the same time points (VanderWeele, 2019) and avoiding some accepted limitations of retrospective reporting of ACEs, including memory decay (Hardt & Rutter, 2004), recall bias (Manyema & Richter, 2019) and mood-congruent bias (Wolkind & Coleman, 1983). However, the prospective reporting of ACEs is not without limitations, including the fact that the developmental period of childhood was not yet over at the time of data collection. Subsequently, ACEs experienced after the data collection had ceased but still happened before 18 years old may have been missed and there may be an under-reporting of ACEs. This may mean that the 5 classes reflect different patterns of ACEs but also may reflect different patterns in who reported what experiences. Furthermore, the data are over 10 years old and whilst these results may not be as generalizable to present day adolescents, our discussion with social workers practising in 2024 confirmed the results still have relevant practice implications. Furthermore, the samples were recruited from low-resource and high deprivation contexts similar to where many adolescents in South Africa grew up, and subsequently the results may be applicable to other South African settings.

In binary scoring the ACEs, we limited our opportunity to investigate how specific experiences combined. For example, for sexual abuse, we missed an opportunity to look at specific probabilities of items such as experiencing unwanted touching of private parts versus forced sex. In creating dichotomous variables, we also limited our ability to explore the severity and frequency of each experience and in doing so, we may have missed nuance in children’s experiences of ACEs. Future research could implement person-centred approaches to ACEs whilst also exploring the frequency and severity of ACEs.

We are aware the context in which this study is set in has a sizeable number of children cared for by people other than their parents due to high rates of parental mortality. Therefore, our parental morbidity variable may have not been as relevant of adverse childhood experience for those children living with caregivers other than biological parents. The way the questions were structured unfortunately did not allow us to ascertain caregiver morbidity. Interviewers defined the term “sick” to the child participants as people who are ill over a long period or who must take regular medication. Whilst sickness was defined to mitigate the reporting of short-term illness such as a common cold, this definition still may have introduced variability in how children interpreted and reported illness. Subsequently, whilst this item captured children’s subjective perceptions of illness in the household, it likely reflects heterogeneous experiences. To account for this limitation, we included a separate indicator of parental AIDS-affectedness based on specific symptoms and diagnoses to more accurately reflect serious, chronic parental illness in this context.

## Conclusion

The current study highlights high ACE exposure in this sample of South African adolescents, with particularly high exposure to contextually sensitive adversities such as witnessing and experiencing community violence, AIDS-related stigma, parental AIDS-affectedness, food insecurity, parental morbidity, and parental mortality. Classes 1 and 4 presented a case-study example of the compounding effect parental AIDS-affectedness had on the likelihood of experiencing other ACEs, confirming not only that ACEs cannot be viewed as additive, but person-centred approaches to ACEs such as LCA are imperative for understanding the complexity and multiplicative effects of ACEs in low-resource settings.

## Supplementary Information

Below is the link to the electronic supplementary material.

Supplementary file1 (DOCX 29 KB)

## Data Availability

Data are available upon request via the UK Data Service (https://beta.ukdataservice.ac.uk/datacatalogue/studies/study?id=851277).
