# Supplementary material for: Patterns of Adverse Childhood Experiences from a Longitudinal South African Community Sample: A Latent Class Analysis
Source: Int J Child Maltreat. 2025 Aug 28;8(4):403–36. doi: 10.1007/s42448-025-00231-5 (PMC12644135; doi:10.1007/s42448-025-00231-5)
Supplement: Supplementary file 1 — Supplementary file1 (DOCX 29 KB) [file 42448_2025_231_MOESM1_ESM.docx]

Supplementary Figure 1: Scree plot of latent class analysis solutions with different numbers of classes according to Bayesian Information Criterion values

Supplementary Figure 2: The 4-class model of ACEs and response probabilities for the 14 ACEs for each class

*Note:* “CV” = Community Violence.

Supplementary Figure 3: The 6-class model of ACEs and response probabilities for the 14 ACEs for each class 4 class solution

*Note:* “CV” = Community Violence.
